# YOLO-based segmented dataset for drone vs. bird detection for deep and machine learning algorithms

**DOI:** 10.1016/j.dib.2023.109355

**Published:** 2023-06-27

**Authors:** Shishir Kumar Shandilya, Aditya Srivastav, Kyrylo Yemets, Agni Datta, Atulya K. Nagar

**Affiliations:** a School of Computing Science and Engineering, VIT Bhopal University, India; b Department of Artificial Intelligence, Lviv Polytechnic National University, Lviv, Ukraine; c Liverpool Hope University, United Kingdom

**Keywords:** Computer vision, Drones vs birds, Drone detection, Deep learning, Machine learning, Image segmentation, Drone security

## Abstract

The use of unmanned aerial vehicles (UAVs) has been rapidly increasing in both professional and recreational settings, leading to concerns about the safety and security of people and facilities. One area of research that has emerged in response to this concern is the development of detection systems for UAVs. However, many existing systems have limitations, such as detection failures or false detection of other aerial objects, including birds. To address this issue, the development of a standard dataset that provides images of both drones and birds is essential for training accurate and effective detection models. In this context, we present a dataset consisting of images of drones and birds operating in various environments. This dataset will serve as a valuable resource for researchers and developers working on UAV detection and classification systems. The dataset was created using Roboflow software, which enabled us to efficiently edit and manipulate the images using AI-assisted bounding boxes, polygons, and instance segmentation. The software supports a wide range of input and output formats, making it easy to import and export the dataset in different machine learning frameworks. To ensure the highest possible accuracy, we manually segmented each image from edge to edge, providing the YOLO model with detailed and accurate information for training. The dataset includes both training and testing sets, allowing for the evaluation of model performance and accuracy. Our dataset offers several advantages over existing datasets, including the inclusion of both drones and birds, which are commonly misclassified by detection systems. Additionally, the images in our dataset were collected in diverse environments, providing a wide range of scenarios for model training and testing. The presented dataset provides a valuable resource for researchers and developers working on UAV detection and classification systems. The inclusion of both drones and birds, as well as the diverse range of environments and scenarios, makes this dataset a unique and essential tool for training accurate and effective models. We hope that this dataset will contribute to the advancement of UAV detection and classification systems, improving safety and security in both professional and recreational settings.

**Specifications Table**

**Table:** 

Subject	Computer Science Computer Vision and Pattern Recognition
Specific subject area	The dataset provides segmented image files of drones and birds for computer vision and pattern recognition applications.
Type of data	Segmented Images, JPEG files for images, Plain Text files for annotations, .xlsx file for reference link of videos and YOLOv7 PyTorch
How the data were acquired?	The dataset consists of 42 different bird and drone videos. A video with the following resolutions was used to extract the frames: 4096 *×* 2160 @25frames per second, 3840 *×* 2160 @30frames per second, 1080 *×* 1920 @25frames per second, and 1280 *×* 720 @60frames per second. These video clips, which last for about a minute, include one or more drones and/or birds in the scene under different lighting and scenarios. The video frames are typically extracted in JPEG format using roboflow.
Data format	Raw, Pre Processed, Filtered
Description of data collection	The images in the dataset are segmented and saved in the YOLOv7 PyTorch format. YOLOv7 is the first from his family, which has new model heads allowing for key points, instance segmentation, and ob- ject detection in segmented images. The images are divided into pixel groupings, which can then be labelled and classified. By providing the exact outline of the object within an image, it is easier to ana- lyse any object, regardless of its distance. Three sectionsTrain, Valid, and Testwere created in the dataset. Since there weren't many drone images available, the dataset here was created by extracting video frames. Before segmentation and augmentation, the video's frames were extracted in roboflow after being downloaded from Pexels web- site www.pexels.com, which provides free stock photos, royalty-free images, and videos shared by creators.
Data source location	• Institution: VIT Bhopal University, Bhopal • City/Town/Region: Bhopal • Country: India • Primary raw sources are available at https://data.mendeley.com/datasets/6ghdz52pd7/5
Data accessibility	Repository name: Mendeley data Data identification number: 10.17632/6ghdz52pd7.5 Direct URL to data: https://data.mendeley.com/datasets/6ghdz52pd7/5

## 1 Value of the Data

• The presented dataset comprises processed RGB photos of drones and birds captured in diverse lighting conditions. This dataset can be utilized for various applications, such as image processing, image segregation, machine learning, and deep learning, to detect and distinguish drones from birds.
• This dataset can be employed to compare and contrast the outcomes of real-time experiments involving drones and birds. It enables the detection and differentiation of multiple drones from birds in different scenarios solely through image processing and analysis.
• The dataset can be utilized to develop a robust drone detection system, which can aid in preventing potential security risks in prohibited areas like airports or crowded places. This system can contribute to enhancing the security of people and facilities by accurately identifying drones and distinguishing them from birds.

## 2 Objective

Due to reduced costs and improved UAV technology, the use of drones is growing in multiple applications, which has led to the identification of drones as a vital object detection challenge [2], [3]. However, detecting distant drones in adverse situations, such as low contrast, long-range, and limited visibility, necessitates the use of effective identification algorithms. However, not only does the machine learning algorithm need to be effective, but it also requires a large dataset to train it to detect and identify drones and birds in diverse environments [4], [5], [6]. The dataset presented in this paper was produced by extracting frames from 42 different videos, under different lighting conditions and scenarios.

## 3 Data Description

A total of 42 videos were utilized for making this dataset, including both drone and bird videos. The footage used to extract the frames has a resolution of:

• 4096 *×* 2160 @25 frames per second,
• 3840 *×* 2160 @30 frames per second,
• 1080 *×* 1920 @25 frames per second,
• 1280 *×* 720 @60 frames per second.

These clips are generally a minute long and feature one or more drones or birds in the frame. This video was obtained from the Pexels website, and its frames were extracted in roboflow before segmentation and augmentations. Apart from the video frame extraction, a few bird images were also captured with a digital camera at the VIT Bhopal University in India, where a large variety of birds are sighted. To improve the performance of the models that will be using this dataset, augmentation has been applied to increase the diversity of the cases in the dataset.

In the presented dataset [1], every image file has its own accompanying text file in YOLO format. If no object is found, then the given algorithm will just skip over the image. In the text file, the following information can be found in the following order: (*class_ID,×_centre, y_centre, width, height*).

The dataset has been meticulously organized into three distinct folders, each designated for training, validation, and testing, with further division into two subfolders to store images and corresponding labels. This comprehensive dataset comprises a vast array of 20,925 images, including 8451 images of birds and 12,474 images of drones, all of which have been meticulously compressed to achieve an optimal size of 640 *×* 640 pixels and saved in the widely popular JPEG format. The strategic choice of adopting the YOLO format, which utilizes a one-level detection architecture, enables real-time detection with ease, as YOLO provides superior processing times and faster frame rates compared to two-level detection architectures, making it the go-to tool for object detection tasks across various industries.

The one-level detection architecture implemented by YOLO offers notable advantages over traditional two-level detection architectures. Unlike two-level architectures that require multiple passes through the network to detect objects, YOLO accomplishes this in a single, efficient pass, significantly reducing the computational cost, which is particularly advantageous for real-time applications where speed and accuracy are critical. Moreover, YOLO demonstrates exceptional accuracy in detecting small ob jects, surpassing two-level architectures that often struggle with smaller objects due to multiple stages of classification. The choice between YOLO and other two-level detection architectures depends on factors such as object size, image complexity, and desired trade-offs between detection speed and accuracy.

The presented dataset stands out from other publicly available datasets due to its meticulous segmentation of objects, namely birds and drones. Each frame containing a detected bird or drone has undergone a meticulous process of segmenting the ob ject from the background, resulting in a highly precise representation that can significantly augment the algorithm's detection capabilities in real-time footage. This segmentation approach provides superior accuracy and granularity compared to simple bounding box annotations, as it captures the exact shape, size, and location of the objects at the pixel level.

The availability of a segmented dataset is particularly valuable in tasks that demand precise localization of objects, such as the detection of birds and drones in real-time footage. The meticulous segmentation in this dataset enables the algorithm to gain a refined comprehension of the visual features of the objects, leading to improved detection accuracy and performance.

Additionally, the presented dataset incorporates mosaic subparts in the train folder images, which serves as a crucial component in enhancing the algorithm's capability to detect diminutive objects, such as birds or drones, that may be located at a considerable distance from the camera source in the frame. By dissecting the images into smaller, finely detailed subparts, the mosaics provide an enhanced resolution and focused view of these objects, enabling the algorithm to discern their intricate visual attributes and acquire the acumen to accurately detect them.

The inclusion of mosaics in the training process creates a realistic training environment where the algorithm is exposed to diverse scenarios involving smaller objects dispersed across different regions of the image, even those situated at a significant distance from the camera source. This allows the algorithm to develop proficiency in discerning and precisely localizing diminutive objects in real-time footage, despite the inherent challenges posed by their reduced size and low contrast. By leveraging the unique feature of mosaic subparts in the training process, the algorithm gains a substantial advantage in developing a robust capability to effectively detect smaller objects, such as birds or drones, even when they are located at a considerable distance, thereby establishing the presented dataset as a valuable resource for advancing object detection prowess in challenging scenarios.

## 4 Experimental Design, Materials and Methods

### 4.1 Materials

To create our dataset of drone and bird images for real-world detection using YOLO, we utilized Roboflow software. Roboflow allowed us to efficiently edit and manipulate the dataset, using AI assistance for bounding boxes, polygons, and instance segmentation. By using publicly accessible models, we were able to automatically annotate images, saving time and effort. Robo- flow offers a range of input formats such as JPG, PNG, BMP, MOV, MP4, and AVI, which facilitates the easy importation of the necessary images for dataset creation.

The software also allows for easy export in many different formats, including coco JSON, VGG, Vott JSON, Marmot XML, YOLO PyTorch, YOLO Darknet TXT, and Kaggle CSV, making it simple to use the data in a variety of machine learning frameworks. One of the major advantages of using Roboflow was its intuitive interface and intelligent defaults. This allowed us to annotate images quickly and accurately, without the need for extensive training or specialized knowledge. Additionally, Roboflow has strict privacy and security protocols, ensuring that our data was safe and protected.

To ensure the best possible results, we manually segmented each image from edge to edge, ensuring that the YOLO model would have accurate and detailed information to work with. This attention to detail and focus on accuracy will ensure that our model can accurately detect and classify drones and birds in real-world scenarios. By utilizing the advanced features of Roboflow and taking the time to manually segment each image, we were able to create a high-quality dataset that will enable our model to perform at its best, even in challenging environments.

**Fig. 1:**
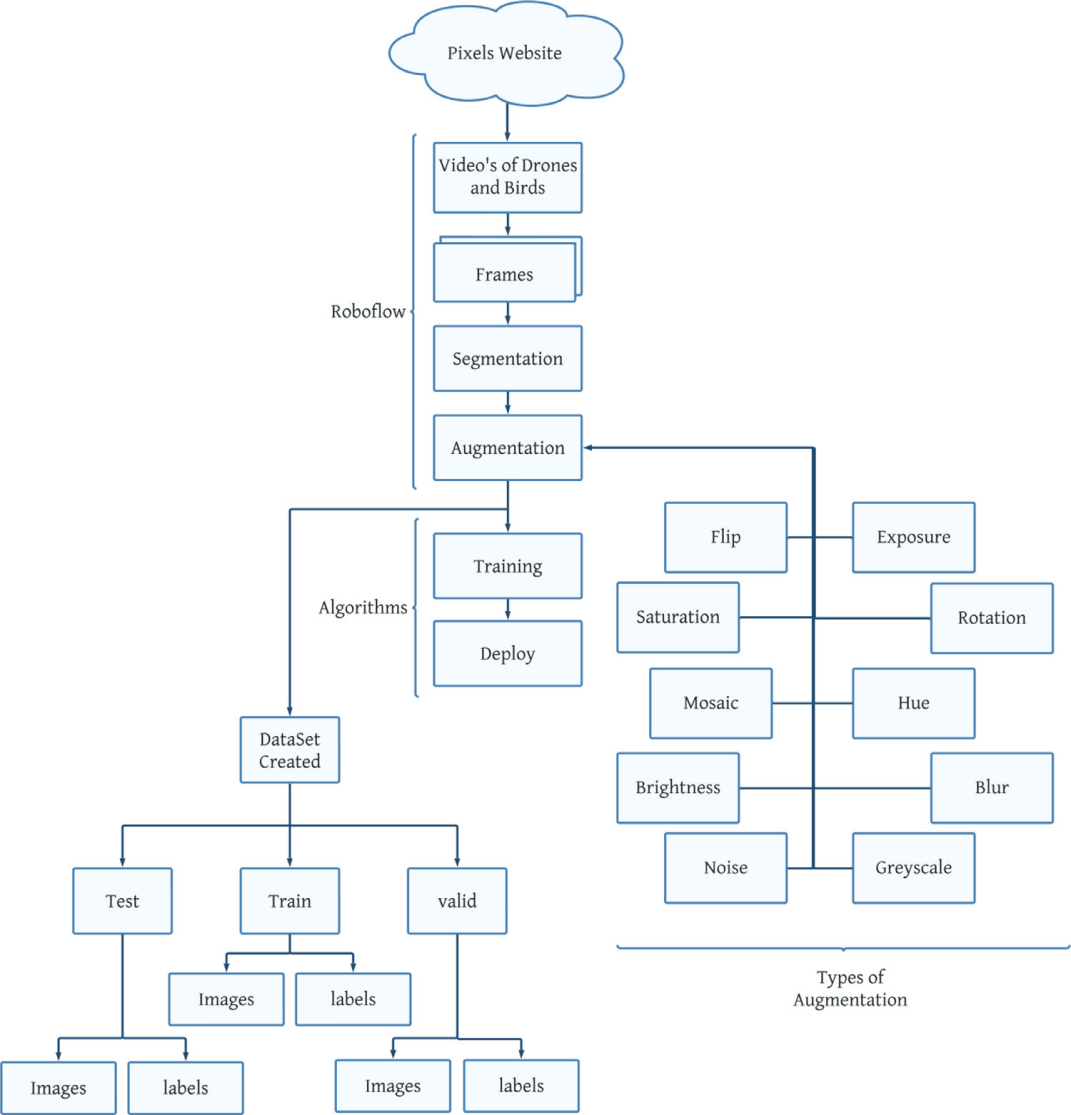
Flowchart of creating the DataSet.

In Fig. 1, the entire process of dataset creation is presented, showcasing the sequential steps involved in generating a dataset for a specific task or research study. The figure provides an overview of how data is collected from diverse sources, undergoes pre- processing to ensure quality and usability, and goes through annotation and labeling processes. It also illustrates the partitioning of the dataset into training and validation sets, along with the application of data augmentation techniques to increase diversity. The final dataset obtained after these stages is ready for utilization in training machine learning models, conducting statistical analyses, or further data-driven research endeavors.

### 4.2 Methods

Prior to downloading the videos in various resolutions from the pexels website, each video was categorized and listed on a spread- sheet. Images were later extracted from the videos frame by frame, imported into the roboflow, and saved. Later, all the images were annotated. Each image had to be manually segmented to avoid any mistakes that might occur in the automated annotation. At the beginning of the annotation, two classes were created: zero for drones and one for birds. It is added to the dataset, preprocessed, and cleaned using roboflow in YOLO format. Prior to training the model, all images must be standardized, which involves altering the size, orientation, or color as per the requirement.

The generated dataset has been cleaned up and pre-processed, which allows the algorithms to use the images as input without any further preprocessing to save time. This dataset has been preprocessed with the YOLO format, but some preprocessing and cleaning are still necessary to meet the requirements of the selected model. The most important aspect of preprocessing is resizing, due to which all the images are resized to 640 *×* 640 pixels while being added to the dataset. Considering that all the images are the same size, the algorithms will run more quickly during training and inference. The convolutional neural networks will also support the images using fully connected layers because the images are of equal size.

**Fig. 2:**
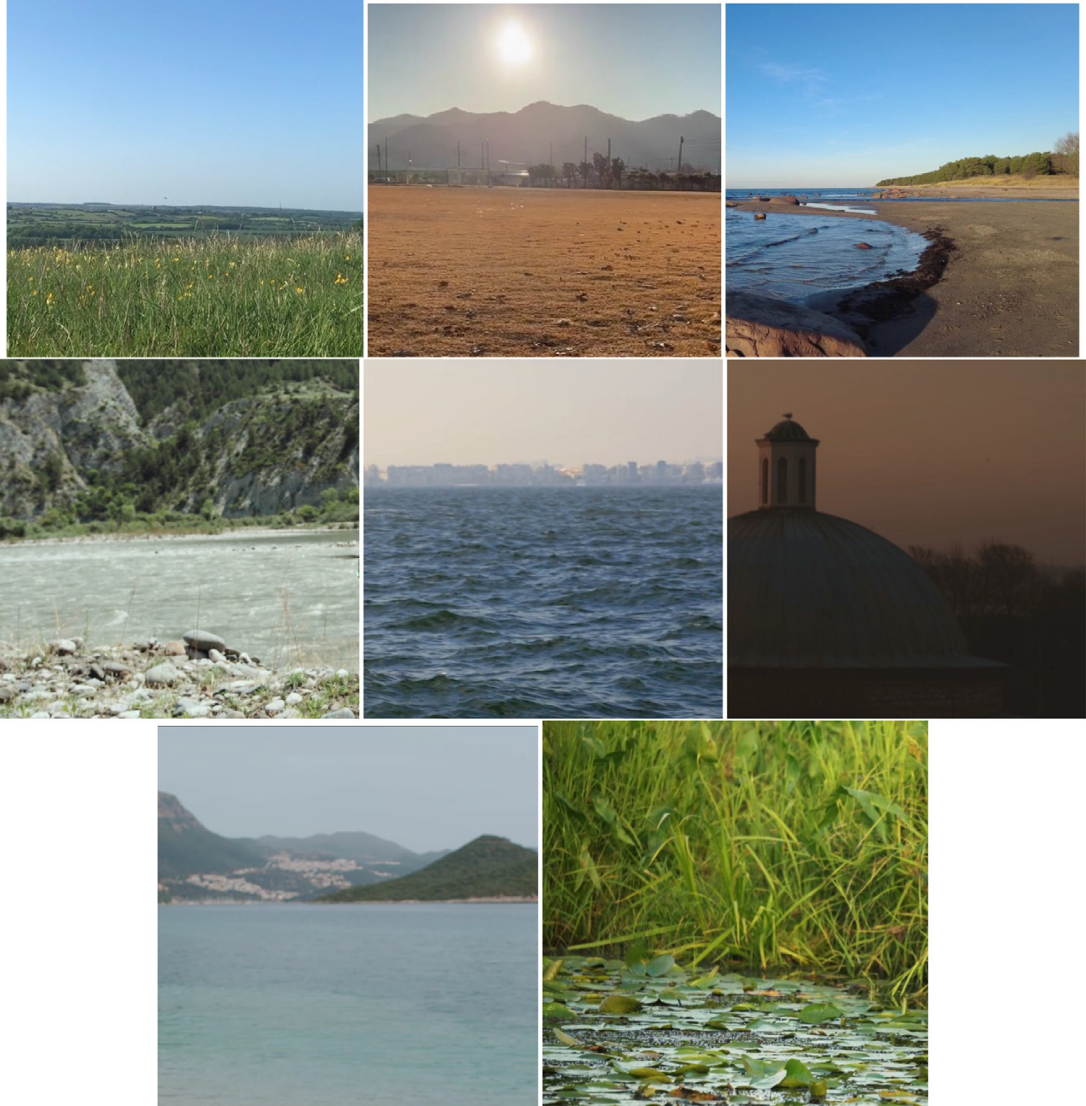
Diverse Scenario Examples.

In Fig. 2, several instances of scenarios are presented as examples.

As per Table 1, to enable image reading from multiple viewpoints, random flips are applied in addition to left-to-right or up- to-down reading. To enhance algorithmic variety, the angle for rotation is set to approximately 34°. This augmentation technique helps the algorithm to learn from diverse image orientations and viewpoints, thereby improving its ability to generalize to different image conditions. Because the dataset contains a variety of conditions, few scenarios have multiple lighting situations. As mentioned in Table 1, random exposure is applied to account for various lighting scenarios in the dataset. This augmentation technique is crucial in assisting the algorithms in generalizing and learning to perform tasks in different lighting conditions. When the algorithms are overfitting on the wrong thing or producing false positives, noise plays a crucial role in enhancing these conditions. The method used in this dataset, known as ``salt and pepper,'' converts random portions of a pixel's color into either black or white. Noise can be implemented in a variety of ways.

**Table 1:** Augmentation table.

Rotation	Between -43°and +43°	Added variability to rotations to help the model be more resilient to camera roll.
Greyscale	Apply to 25% of Images	Probabilistically apply greyscale to a subset of the train
		ing set.
Hue	Between -60°and +60°	Randomly adjust the colours in the image.
Saturation	Between -42% and +42%	Randomly adjust the vibrancy of the colours in the im
		ages.
Brightness	Between -34% and +34%	Added variability to image brightness to help the
		model be more resilient to lighting and camera setting
		changes.
Exposure	Between -17% and +17%	Added variability to image brightness to help the
		model be more resilient to lighting and camera setting
		changes.
Blur	Up to 4.25 pixels	Added random Gaussian blur to help the model be
		more resilient to camera focus.
Mosaic	-	Added mosaic to help the model perform better on
		small objects.
Bounding Box: Brightness	Between -25% and +25%	Added variability to annotated image brightness to help
		the model be more resilient to lighting and camera set-
		ting changes.
Bounding Box: Exposure	Between -25% and +25%	Added variability to annotated image brightness to help
		the model be more resilient to lighting and camera set-
		ting changes.
Bounding Box: Blur	Up to 8 pixels	Added random Gaussian blur on annotated images to
		help the model be more resilient to camera focus.
Bounding Box: Noise	Up to 5% of pixels	Added noise to help the model be more resilient to cam
		era artefacts.

Resizing is one of the most important stages of pre-processing. Given that CNNs’ fully connected layers demand that all the images have equal-sized arrays, this speeds up both training and inference. By preventing the model from overfitting to specific spots in the images given as input, rotation is a crucial augmentation technique that ensures the model is neutral regarding the orientation of the inputted images. The three matrices R (red), G (green), and B (blue) are used to represent colors, while a single matrix with a black-to-white range is used to represent grayscale. The step of converting to grayscale can be applied to both the training and test sets because it significantly affects the computational speed.

For better accuracy in algorithm training and deployment, random flips are employed. Random exposure is applied as there are various lighting scenarios; this step is crucial for helping the model generalize so that it can learn to perform the task in various lighting scenarios.

**Fig. 3:**
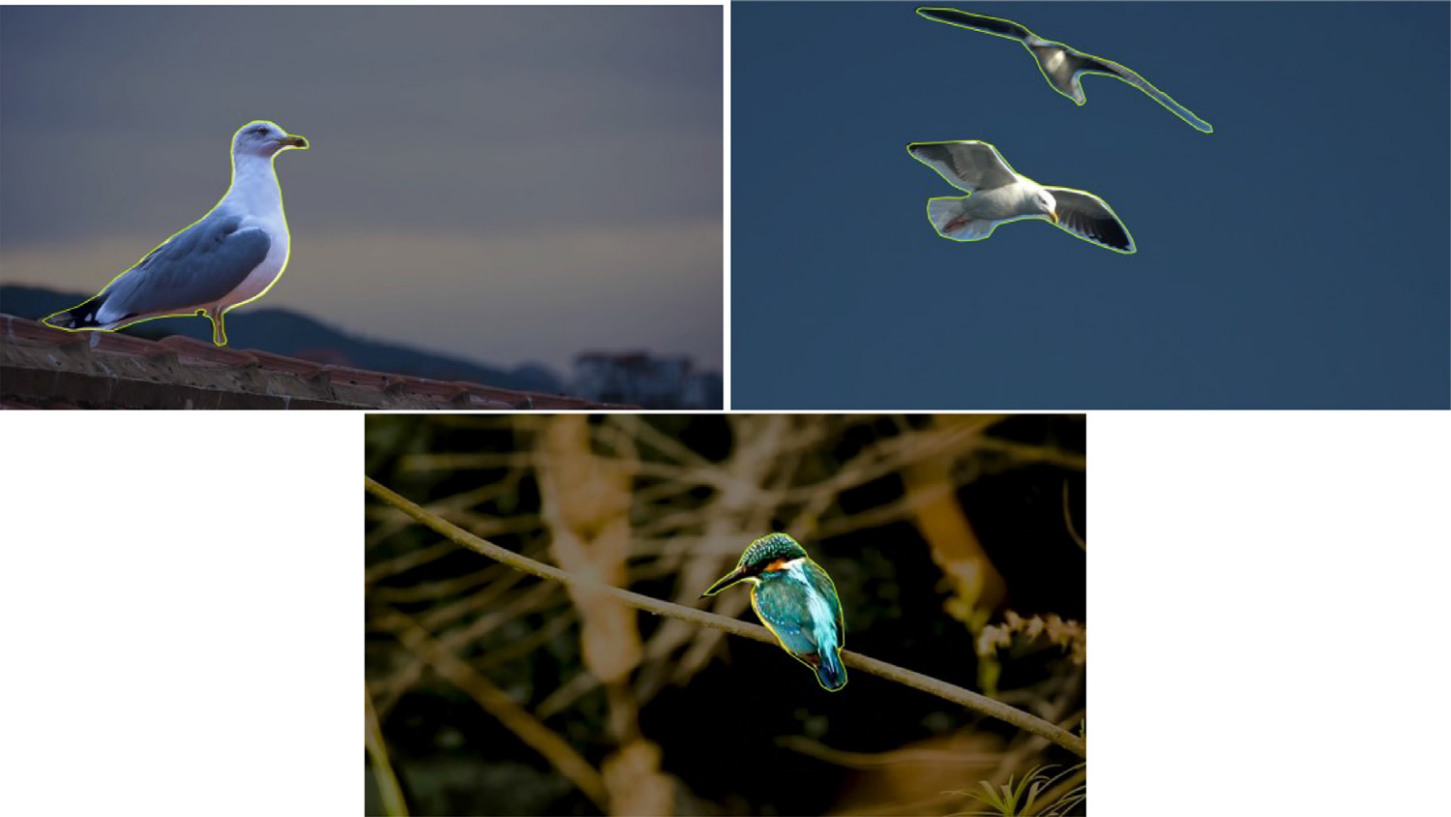
Sample Images of Bird.

**Fig. 4:**
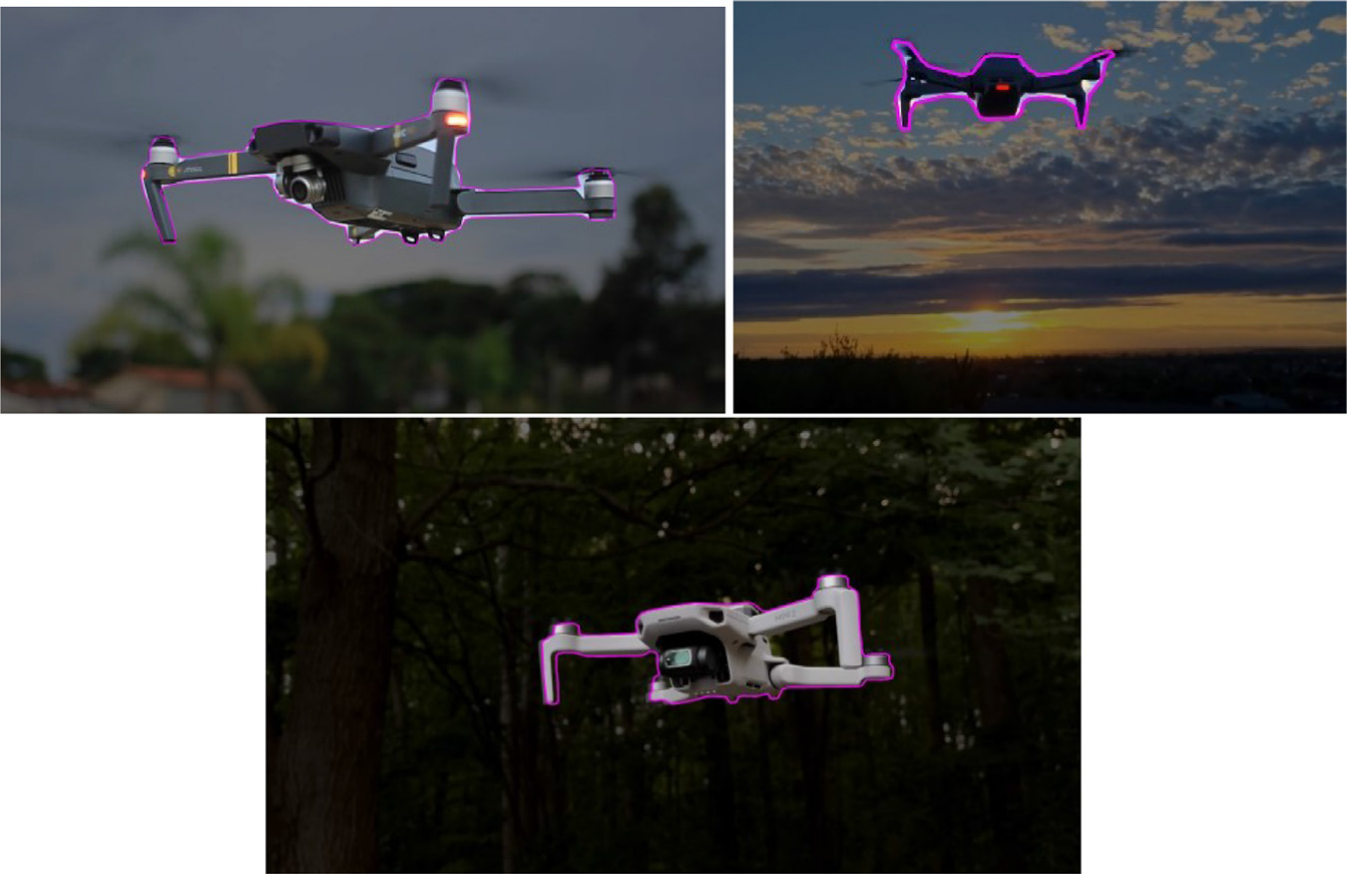
Sample Images of Drone.

A few examples of segmented bird and drone images are given in Figs. 3 and 4:

The dataset is generated in YOLOv7 PyTorch format. The images must be categorized, tagged, segmented, and annotated in order for computers to recognize the objects of interest in these images. This will help the models to understand broad scenarios and to enable deep learning or machine learning. Semantic segmentation is applied in image annotation to assist in recognizing items in a single class. The dataset is intended to be used for testing and training algorithms for in-flight drone and bird detection from any camera module or from the ground.

**Fig. 5:**
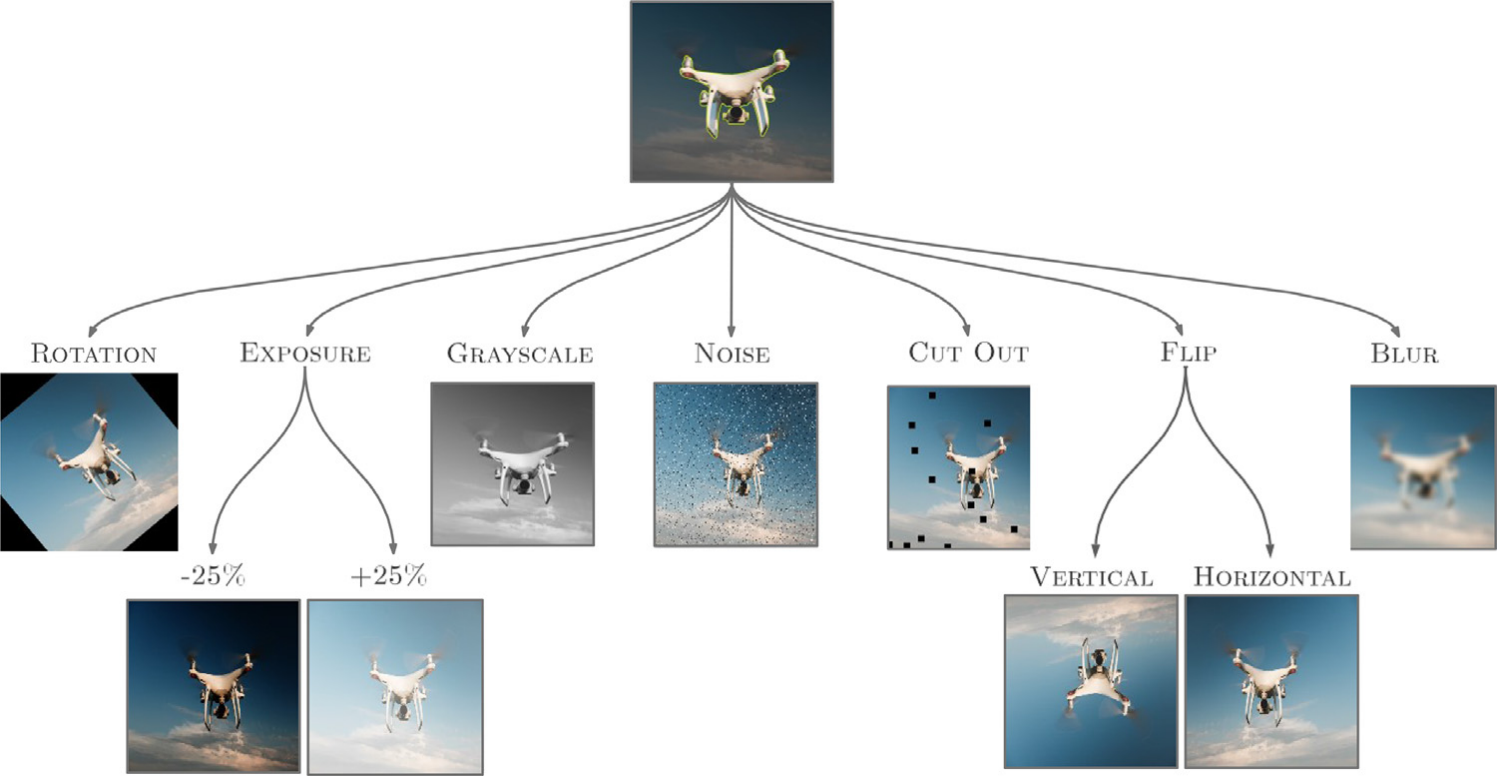
Augmentation visual examples with drone images.

In Fig. 5, different types of augmentation techniques are showcased to demonstrate the variety of transformations applied to drone images. The purpose of this figure is to illustrate the range of augmentation possibilities, including rotation, exposure adjustment, grayscale conversion, noise addition, blur effect, and flipping. Each augmentation technique serves as a visual representation of the specific transformation without providing detailed explanations or specific applications. The figure aims to provide a quick overview of the types of augmentations used in the context of drone image processing.

## Ethics Statements

The proposed work does not require the collection of any data from social media platforms. Instead, we sourced images from pexels.com, which is a platform that distributes copyright-free content. To ensure compliance, we have reviewed the data redistribution policies available at https://www.pexels.com/terms-of-service/.

The authors confirm that the provided data set and presented work strictly meet the ethics requirements for publication in Data in Brief as mentioned in https://www.elsevier.com/authors/journal-authors/policies-and-ethics.

## CRediT authorship contribution statement

**Shishir Kumar Shandilya:** Conceptualization. **Aditya Srivastav:** Data curation, Methodology. **Kyrylo Yemets:** Writing – original draft, Visualization, Investigation. **Agni Datta:** Methodology, Formal analysis. **Atulya K. Nagar:** Conceptualization.

## Declaration of Competing Interest

The authors declare that they have no known competing financial interests or personal relationships that could have appeared to influence the work reported in this paper.

## Data Availability

Segmented Dataset Based on YOLOv7 for Drone vs. Bird Identification for Deep and Machine Learning Algorithms (Original data) (Mendeley Data) [1].
